# Pharmacokinetic Study of Intranasal Dexamethasone and Methylprednisolone Compared with Intravenous Administration: Two Open-Label, Single-Dose, Two-Period, Two-Sequence, Cross-Over Study in Healthy Volunteers

**DOI:** 10.3390/pharmaceutics15010105

**Published:** 2022-12-28

**Authors:** Graciela Cárdenas, Raúl J. Bobes, Gladis Fragoso, Nicolas I. Pérez-Osorio, Marisela Hernández, Alejandro Espinosa, Agnes Fleury, José Flores, The Revival Project Consortium, Juan Pedro Laclette, Edda Sciutto, Helgi Jung-Cook

**Affiliations:** 1 Departamento de Neurología, Instituto Nacional de Neurología y Neurocirugía, Av. Insurgentes Sur 3877, La Fama, Tlalpan, Mexico City 14269, Mexico; 2 Instituto de Investigaciones Biomédicas, Universidad Nacional Autónoma de Mexico, Coyoacán, Mexico City 04510, Mexico; 3 Facultad de Química, Universidad Nacional Autónoma de Mexico, Coyoacán, Mexico City 04510, Mexico

**Keywords:** dexamethasone, methylprednisolone, intranasal, neuroinflammation, pharmacokinetics

## Abstract

Dexamethasone (DXM) and methylprednisolone (MEP) are potent glucocorticoids used to control several inflammatory conditions. Evidence of delayed DXM reaching the central nervous system (CNS) as well as tachyphylaxis and systemic, undesirable side effects are the main limitations of peripheral delivery. Intranasal administration offers direct access to the brain as it bypasses the blood–brain barrier. The Mucosal Atomization Device is an optimal tool that can achieve rapid absorption into the CNS and the bloodstream across mucosal membranes. This study was designed to evaluate and compare the bioavailability of DXM and MEP after intranasal versus intravenous administration. Two open-label, balanced, randomized, two-treatment, two-period, two-sequence, single-dose, crossover studies were conducted, which involved healthy male and female adult volunteers. After intranasal administration, DXM and MEP were detected in plasma after the first sampling time. Mean peak concentrations of DXM and MEP were 86.61 ng/mL at 60 min and 843.2 ng/mL at 1.5 h post-administration, respectively. DXM and MEP showed high absolute bioavailability, with values of 80% and 95%, respectively. No adverse effects were observed. DXM and MEP systemic bioavailability by intranasal administration was comparable with the intravenous one, suggesting that the intranasal route can be used as a non-invasive and appropriate alternative for systemic drug delivery.

## 1. Introduction

Dexamethasone (DXM) and methylprednisolone (MEP) are synthetic glucocorticoids that are widely used in clinical practice to modulate several inflammatory conditions [1,2,3,4,5]. They are usually administered through the intravenous (IV), intramuscular, and oral routes as anti-inflammatories and immunosuppressants. DXM has also been administered intranasally at low doses to treat allergic conditions, aiming to reach higher local levels and minimize negative side effects [6]. MEP has been tested intranasally only in experimental models of inflammation [7]. DXM and MEP have also been used to control neuroinflammation in several neurological diseases such as multiple sclerosis, brain tumor-associated edema, and neurocysticercosis, among others [8,9,10,11,12].

Although both corticosteroids are liposoluble drugs that may passively diffuse through the cell membrane [13], several intrinsic and extrinsic factors influence the CNS penetrance, such as the molecular weight, the lipophilicity, the number of hydrogen bonds, and the presence of drug transports (P-glycoprotein, P-gp). Among them, particular attention has been paid to the multidrug resistance transporter P-gp due to its important role in the CNS penetration of highly lipophilic molecules from the blood [14], and which consequently regulates the intracellular levels of glucocorticoid hormones and the function of the glucocorticoid receptor, especially in response to antidepressant drugs [15].

The P-gp is highly and constitutively expressed in the liver, intestine, kidney, pancreas, adrenal, capillary endothelium (BBB), the blood–testis barrier, choroid plexus, placental trophoblast, and others [16,17]. This transporter captures substrates from the inner leaflet of the lipid bilayer of the cell membrane, entering by passive diffusion; they are later pumped out, thus preventing them from being accumulated in the cells. In the case of CNS, P-gp reduces brain penetration after the intranasal administration of glucocorticoids [18]; thus, pharmacokinetic studies in systemic blood cannot directly extrapolate what occurs in the CNS [19].

Until now, there is no study on nasal administration of DXM and MEP with CNS penetration involving healthy volunteers. However, there are some reports on multiple sclerosis and spinal cord injury that account the arrival of MEP in the CNS [20,21]. In addition, in mice, higher levels of DXM have been observed in different regions of the brain after intranasal versus intravenous administration soon after being administered [22].

To improve the efficacy and safety of corticosteroids, different strategies have been proposed to prevent their rapid elimination and to provide targeted and controlled release [23,24]. Among them, GC conjugation with natural polymers, lipid particles, dendrimers, micelles, liposomes, or implants, which has accounted for a better control of their release, increases the permanence of the drug at the target site, thereby reducing the frequency of the doses and the risk of side effects [23,24].

In the early 1990s, the efficacy of the intranasal (IN) route to directly deliver drugs to the central nervous system for therapeutic purposes was clearly evidenced [25]. Since then, numerous studies have demonstrated the extensive therapeutic options offered by this administration route. Recently, we demonstrated that IN drug administration allows for the CNS to be reached in minutes, even after low-dose administration [22], as well as its efficiency in controlling the neuroinflammatory response accompanying different experimental neuropathologies in murine models. In this context, the IN delivery of glucocorticoids was more efficient than the IV route in controlling neuroinflammation in lipopolysaccharide-induced sepsis [26], autoimmune experimental encephalitis [27], ischemic stroke [28], and chronic toluene exposure [29]. Likewise, the olfactory and trigeminal nerves that innervate the nasal cavity provide a direct connection of the nasal epithelium with the CNS; this, coupled with the extensive vascularization of the mucosa and the lamina propia as well as the permeable nature of this epithelium, yields an optimal absorption surface for IN drug delivery, for central as well as for systemic effects. On top of that, nasal drug administration offers the possibility of reaching active levels in the respiratory system in about one minute after administration [30]. In this context, the IN route has been successfully employed to control systemic inflammation in a mouse model for pulmonary tuberculosis [31].

Since the onset of the COVID-19 pandemic, the need for a drug treatment to control systemic inflammation [32,33] was promptly identified. Only several months after the beginning of the pandemic, a study carried out on thousands of patients showed that low doses of DXM in hospitalized patients requiring respiratory support (from supplemental oxygen to mechanical respiration) significantly reduced mortality [34]. In the same line, it was demonstrated that MEP was useful for preventing mechanical ventilation in COVID-19 pneumonia [35], decreasing mortality even more than DXM [36,37,38]. Despite the low doses of corticosteroid used, further studies have shown some unwanted side effects. In fact, a growing number of reports on emerging systemic fungal infections concomitant with COVID-19 has been published [39,40,41,42,43], raising concerns about the risk of immunosuppression due to both corticosteroids, particularly among the high-risk populations (diabetes, obesity, and aging).

As the pandemic evolved, it has been extensively documented that the SARS-CoV-2 virus gains access to multiple CNS cell types expressing the ACE2 receptor, triggering neuroinflammatory reactions [44,45]. However, an exacerbated peripheral inflammation can disrupt the BBB through several pathways, increasing the neuroinflammation originally triggered by the viral infection on CNS cells [46].

In this context, considering the scarce pharmacokinetic data on IN DMX and MEP, we aimed to evaluate the pharmacokinetics and comparative bioavailability of these drugs IN (using the MAD nasal device) versus IV in healthy volunteers.

## 2. Materials and Methods

### 2.1. Study Design

Two open-label, balanced, randomized, two-treatment, two-period, two-sequence, single-dose, crossover studies were conducted involving healthy male and female adult volunteers. Each volunteer received one dose of either DXM or MEP, followed by a one-week elimination (wash-out) period before a new administration of the same medication. Volunteers were randomly assigned to a protocol-specific sequence. The randomization included two treatment sequences. Therefore, each volunteer received DXM or MEP by both routes of administration.

### 2.2. Volunteers

A total of 16 healthy subjects were included (8 women and 8 men), half for the DXM and the others for the MEP study; in each period, the drug was administered intravenously to 4 subjects (treatment A) and intranasally to the other 4 (treatment B), according to a randomization list generated prior to the start of the clinical phase.

Participants eligible for the study were healthy adults aged 23–42 years who demonstrated proper nasal inhalation ability during a screening visit. The clinical screening examination included the individuals’ medical history, physical examination, vital signs, body mass index (≥18 and ≤27 kg/m^2^), weight (>50 kg), and ancillary laboratory tests (blood chemistry, hematology, blood coagulation, and serology). Candidates were excluded when a history of any condition or alteration of the nose or nasal mucosa, including irritation, inflammation, bleeding, excoriation, or ulceration, was detected; individuals with a history of hypersensitivity to the study drug or a history of cardiovascular, renal, hepatic, metabolic, gastrointestinal, neurological, endocrine, hematopoietic conditions (any type of anemia), mental illness, or other organic abnormalities were also excluded. Once enrolled, the volunteers were asked to avoid drinking or using any medication or herbal medicine for at least 14 days; alcohol- and caffeine-containing products were also eliminated for at least 24 h prior to drug administration. The demographic data of the DXM and MEP-administered patients are summarized in Table 1 and Table 2, respectively.

### 2.3. DXM and MEP Dosing

Drug formulations employed were a DXM phosphate injectable solution of 8 mg/2 mL (Alin^®^, Productos Farmacéuticos, Mexico City, Mexico), and an MEP sodium succinate (SOLU-MEDROL^®^ Pfizer, A. de C.V. Aguascalientes, México) injectable solution of 500 mg/8 mL).

Volunteers were randomly assigned to receive a single dose of 1.5 mL DXM (equivalent to 6 mg of dexamethasone) by IV bolus or MEP (an intravenous treatment bolus of 1 mL, equivalent to 62.5 mg of Methylprednisolone), or the same dose intranasally by using a Mucosal Atomization Device (MAD Nasal).

Atomization was performed, with each participant sitting up and with a slight backward head tilt to allow for the optimal spread and absorption of the atomized solutions. No food intake was permitted 10 h before and 4 h after dosing. The nominal doses were similar, allowing for direct pharmacokinetic comparison without dose normalization. Venous blood samples were obtained via an indwelling catheter before administration and at 0.25, 0.5, 0.75, 1, 1.5, 2, 3, 4, 8, 12, and 24 h for DXM and at 0.333, 0.50, 0.667, 0.833, 1, 1.0, 2, 3, 4, 6, 8, 10, 12, and 24 h for MEP after administration. Plasma was separated and frozen at −70 °C for further analysis.

### 2.4. Bioanalytic Method: DXM

DXM concentrations in plasma were determined by reverse liquid chromatography and detected by tandem mass spectrometry (LC-MS/MS). Analysis of samples was performed using an HPLC system Shimadzu SIL-HTA coupled with a turbo ion spray ionization-triple quadrupole mass spectrometer API 4000 (AB MDS Sciex, Toronto, ON, Canada), with positive ion electrospray ionization using the multiple reaction monitoring (MRM) mode. Briefly, 100 µL of internal standard (itopride) and 200 µL of 30% ammonium hydroxide were added to 200 µL of the plasma sample. After vortex mixing for 1 min, a mixture of 3 mL of ether:dichloromethane (70:30) was added. The mixture was vortexed and centrifuged. The organic layer was evaporated to dryness under a stream of nitrogen gas at 40 °C. The residue was reconstituted with 200 µL of the mobile phase, and 10 µL was injected into the system. Separation was achieved using a Zorbax Eclipse Plus^®^ (Agilent Technologies, Mexico City) C_18_ column (3.5 μm, 75 mm × 4.6 i.d.). The mobile phase was composed of 5 mM methanol HPLC ammonium formate in water (95:5 *v/v*) at a flow rate of 0.9 mL/min. This method was fully validated previous to the beginning of the study and was found linear in the range of 5–300 ng/mL. Intra-day and inter-day coefficients of variation were less than 15%. Samples were stable in the autosampler for 24 h. Long-term stability showed that samples were stable for at least 2 months at −50 °C. No matrix effect was observed under the studied conditions.

### 2.5. Bioanalytic Method: MEP

MEP concentrations in plasma were determined by reverse liquid chromatography and detected by tandem mass spectrometry (LC-MS/MS). Analysis of the samples was performed using an HPLC system Agilent G1312C coupled with a turbo ion spray ionization-triple quadrupole mass spectrometer Agilent G6410B, with negative ion electrospray ionization and using the MRM mode. Briefly, 400 µL of acetonitrile was added to 100 µL of the plasma sample. After vortex mixing for 4 min, the samples were centrifuged. The supernatant was separated, and 10 µL was injected into the chromatographic system. Separation was achieved using a Zorbax Eclipse Plus ^®^ (Agilent) C_18_ column (3.5 μm, 100 mm × 4.6 i.d.). The mobile phase was composed of methanol HPLC ammonium formate, 2.5 mM in water (90:10 *v/v*), at a flow rate of 0.8 mL/min.

The method was linear and in the range of 5–1000 ng/mL. Intra-day and inter-day coefficients of variation were less than 15%. Long-term stability showed that the samples were stable for at least 5 months at −60 °C. No matrix effect was observed under the studied conditions.

### 2.6. Pharmacokinetic and Statistical Analysis

The pharmacokinetics of both products was determined by non-compartmental analysis using the Phoenix^®^ WinNonlin^®^ 8.3 Centara L.P. software, Princeton, NJ, USA. The C_max_ and t_max_ were determined by analyzing the concentration profiles vs. time. The determination of the area under the curve, from time zero to the last sampling time (AUC_0–t_), was performed via the trapezoidal rule. The constant of elimination (K_el_) was determined from the linear terminal part of the data that had been logarithmically transformed and was finally estimated through a simple linear regression analysis that considered three different concentrations from the t_max_ value. It was used to determine the area under the curve, from time zero to infinity (AUC_0–∞_), according to the equation: AUC_0–∞_ = AUC_0–t_ + C_t_/K_el_, where C_t_ is the concentration at the last sampling time used. The elimination half-life was determined by the ratio of In (2)/K_el_.

Absolute bioavailability was calculated according to the following equation:

$$F\;\mathrm{abs}={\mathrm{AUC}}_{0-\inf}\;\mathrm{intranasal}/{\mathrm{AUC}}_{0-\infty }\mathrm{intravenous}\;\times \;100\;\frac{{\mathrm{AUC}}_{0-\inf}\;\mathrm{intranasal}}{{\mathrm{AUC}}_{0-\infty }\;\mathrm{intravenous}\;}\times 100$$

### 2.7. Tolerability Assessment of IN Drug Administration

DXM and MEP were easily administered nasally; after administration, the subjects were periodically questioned and monitored for any unusual symptoms. Volunteers were instructed at the beginning of the study to inform the study investigator of any untoward effects (bitter taste, burning sensation, pain) experienced after IN administration. No complications were reported for either the IV or the IN routes of administration. The investigator was responsible for reporting and documenting all adverse events during the study.

## 3. Results

### 3.1. Demographic Data of Volunteers

Volunteers receiving DXM or MEP were divided according to gender: half male and half female. MEP-administered volunteers were 30.75 years old on average and had received 11.25 years of scholarship (Table 1), while the average of DXM administered ones were 30.38 ages old and 13.88 years of scholarship (Table 2). As shown in Table 1 and Table 2, the weight and body mass index (BMI) of both groups were quite similar; in the MEP-administered group, the average values for each parameter were 63.50 kg and 23.75 kg/m^2^, respectively, and for the DXM-administered group, the average values were 64.13 kg and 22.93 kg/m^2^.

### 3.2. Pharmacokinetics Assessment

**Figure 1:**
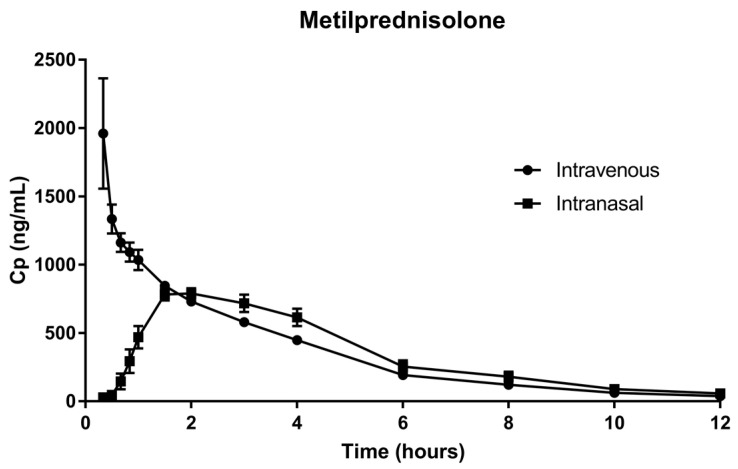
Mean ± SD of the plasma concentration of MEP along time following intravenous or intranasal administration in healthy volunteers.

**Figure 2:**
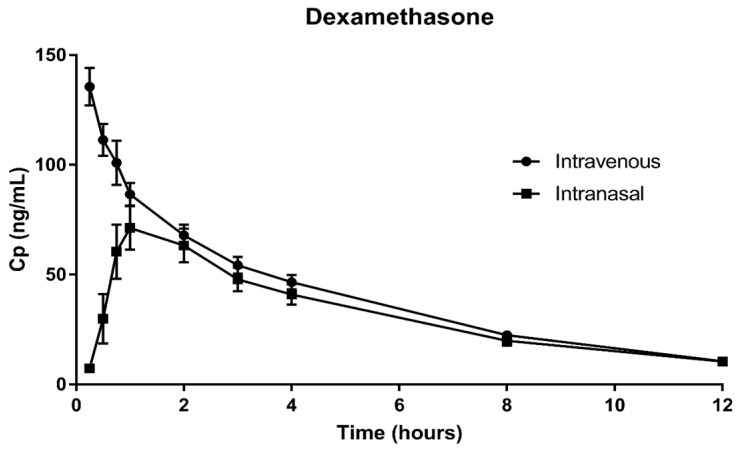
Mean ± SD of plasma concentration of DXM along time following intravenous or intranasal administration.

Figure 1 and Figure 2 show the mean plasma concentration vs. time of the arithmetic data and the semi-logarithmic concentration of DXM and MEP, respectively. Both routes showed the same mean plasma concentrations for DXM at 1.5 h after administration. In the case of MEP, the same mean plasma concentrations were observed after only 1 h of administration. Results of the pharmacokinetic analysis after the IV and IN administration of 6 mg DXM and 62.5 mg MEP to healthy subjects are summarized in Table 3 and Table 4. In both cases, the IV and IN routes resulted in very similar outcomes.

### 3.3. Tolerability Assessment

DXM and MEP were shown to be well-tolerated by both nasal and intravenous routes of administration. In this study, no adverse events were reported.

## 4. Discussion

This study was designed to compare the pharmacokinetics of the intranasal versus the intravenous administration of two of the most widely employed GCs for controlling inflammation: DXM and MEP. Nasal drug administration has been proposed as the most viable alternative to parenteral injections, considering the high permeability of the nasal epithelium, with plasma drug concentration profiles sometimes being equivalent to those obtained via intravenous injection. Our results showed that after intravenous administration, DXM half-life values corresponded with those previously reported [47,48]. In addition, Vd and Cl values were within the ranges reported by Song et al. [49]. To our knowledge, this is the first study to evaluate the pharmacokinetic parameters of nasal administration; the results on IN administration were compared with those reported after extravascular administration. In our study, the between-subject variability was at 18–20%, a value lower than those reported by Queckenberg et al. [50], who found a value of 30% after the administration of DXM in liquid oral solution and through DXM tablets. Toledo et al. [51] evaluated the relative bioavailability and pharmacokinetics of DXM sodium phosphate for injection, which was administered orally. They found that after a dose of 8 mg, a mean Cmax of 79.09 ng/mL was obtained. The Cmax values obtained in the present study were higher than those reported, suggesting that DXM had efficiently been absorbed after nasal administration. In the case of oral bioavailability, the values reported were at 70–78% [52,53]. The bioavailability value of 80% obtained in the present study makes IN DXM an appropriate alternative to IV administration.

The results of MEP showed that after IV administration, the pharmacokinetic parameters obtained agreed with those found in the literature. Cl and Vss values previously reported were at 12–33 L/h and 40–60 L, respectively [54,55]. Recent studies on the pharmacokinetics of MEP after extravascular administration are scarce. Geister et al. [56] evaluated the bioavailability of 8 mg tablets of MEP. They found that the mean tmax value was 2.2 h. Antal et al. [52] evaluated the bioavailability of MEP sodium succinate ester after the intravenous, intramuscular, oral (tablet), or oral solution treatments. They found that the time to reach peak concentration was different for the three extravascular treatments, having absolute bioavailability values of 104, 82, and 75%, respectively. Another study showed that the bioavailability of MEP from a 20 mg tablet was 82% [57]. In the present study, the mean systemic bioavailability of the intranasal MEP administration was 95%, which indicates that the extent of MEP delivered into the general circulation was equal between treatments.

For both GCs, higher levels were detected within the first 2 h after their IV or IN administration. Interestingly, despite individual variability, it was clearly observed that there are no significant differences in the plasma concentration of both drugs two hours after their IN or IV administration. These results agree with data previously reported in experimental mouse studies [22], in which both administration routes were also explored. In the study performed on mice, the concentrations of DXM in brain extracts were also evaluated, demonstrating that in the early stages, the concentration was higher in those who received the steroid intranasally than intravenously. The concentration of both drugs equalized 3 h post-administration. This result disagrees with previous imaging studies carried out on mice, in which a much higher concentration of GCs in the CNS was observed in the animals that received intranasal administration and followed by an analysis 24 h. It is possible that these differences are due to the methods employed to extract and detect the GC in the brains, which did not allow for the recovery and detection of the steroid internalized in the cells [58,59]. On the other hand, the higher intracellular availability of steroid in the CNS when IN-administered may explain its higher efficiency in controlling the neuroinflammation in the different neuropathologies previously reported in experimental models of sepsis, multiple sclerosis, and stroke [26,27,28,29].

It is important to remark that in this study, only the concentrations of both GCs in the blood were evaluated. Thus, this design cannot permit a direct extrapolation to deduce the central nervous system bioavailability. It must consider that although steroid drugs such as DXM and MEP can freely enter into the brain, thus overcoming the BBB [13], other factors may also influence their penetrance into the CNS. In this respect, it has been reported that both drugs are a substrate of the drug efflux transporter P-gp at BBB [19,60], which may limit the steroid levels in the CNS. However, the higher levels of DXM in the CNS in mice that were intranasally versus intravenously administered treatment during the first three hours, along with the higher intracellular level of the steroids in the CNS that remain 24 h after their administration [22], are in accordance with the potential of the IN versus IV/intraperitoneal administration to control neuroinflammation in different experimental models of neuropathologies [26,27,28]. However, to extend the use of this pathway for the control of systemic and central inflammation that accompanies different pathologies in patients, the results of CNS bioavailability studies supporting its use are required. In this context, future studies of pharmacokinetics in the CNS of GC in healthy brains may be performed by positron emission tomography while employing the adequate radiolabeled tracers [61].

Regarding the extent of the GCs that can be reached by the respiratory tract using the IN route, this route can be of particular interest to control inflammatory diseases that affect the respiratory tract [62]. In this respect, an ongoing clinical study on COVID-19 comparing the efficiency of both pathways for dexamethasone administration is showing very promising results [63].

In summary, the results shown in this study allow us to propose the use of IN administration as a route that is as efficient as IV for steroid administration, for the control of systemic inflammation.

## 5. Conclusions

The current study demonstrated that the blood bioavailability of DXM and MEP administered by IN atomization is comparable with that via intravenous administration. The information reported in this study allows us to propose the use of IN atomization as a non-invasive route for the control of systemic inflammation. Further studies are required to confirm the determinant role of different neuropathologies in the control of neuroinflammation.

## Figures and Tables

**Table 1:** Demographic data for DXM-administered patients.

Subject	Gender	Age (Years)	Scholarship (Years)	Weight (kg)	Height (cm)	BMI (kg/m^2^)	Sequence
1	Female	25	9	52	162	19.8	B-A
2	Male	30	17	69	170	23.9	A-B
3	Female	42	12	58	158	23.2	B-A
4	Female	28	17	67	172	22.6	A-B
5	Male	39	17	64	175	20.9	A-B
6	Male	23	16	72	172	24.3	B-A
7	Female	31	9	60	156	24.7	A-B
8	Male	25	14	71	172	24.0	B-A
Average		30.38	13.88	64.13	167	22.93	
Standard Deviation		6.84	3.48	7.0	7.00	1.73	
% CV		22.53	25.10	10.91	4.38	7.55	

Percentage of coefficient of variation (% CV).

**Table 2:** Demographic data for MEP-administered patients.

Subject	Gender	Age (Years)	Scholarship (Years)	Weight (kg)	Height (cm)	BMI (kg/m^2^)	Sequence
1	Female	33	12	67.70	161	26.1	B-A
2	Male	22	6	72.85	175	23.8	A-B
3	Male	35	9	64.50	162	24.6	A-B
4	Male	34	13	62.45	176	20.2	B-A
5	Female	35	14	53.80	152	23.3	A-B
6	Female	32	12	69.80	166	25.3	A-B
7	Female	20	12	44.65	150	19.8	B-A
8	Male	35	12	72.25	164	26.9	B-A
Average		30.75	11.25	63.50	164	23.75	
Standard Deviation		6.14	2.55	9.80	0.09	2.59	
%CV		19.95	22.66	15.44	5.78	10.91	

Percentage of coefficient of variation (% CV).

**Table 3:** Mean pharmacokinetic parameters (sd) for DXM after intravenous and intranasal administration.

Parameter	Intravenous	Intranasal
Cmax (ng/mL)	136.15 (24.77)	86.44 (18.5)
ke h^−1^	0.187 (0.02)	0.183 (0.03)
t_1/2_ h	3.75 (0.5)	3.88 (0.6)
AUC 0-t (ng h/mL)	490.8 (83.6)	376.2 (97.0)
AUC 0-∞ (ng h/mL)	548.2 (92)	430.0 (106.2)
Tmax (h)		
Median		1.0
Minimum		0.75
Maximum		2.0
MRT (h)	4.98 (0.64)	5.8 (0.81)
Cl (L/h/)	10.79 (1.91)	
Vd (L/)	58.37 (11.9)	

**Table 4:** Mean pharmacokinetic parameters (sd) of MEP after intravenous and intranasal administration.

Parameter	Intravenous	Intranasal
Cmax (ng/mL)	1982 (1125.7)	873.55 (105.3)
ke h^−1^	0.33 (0.11)	0.30 (0.03)
t _1/2_ h	2.2 (0.6)	2.3 (0.25)
AUC 0-t (ng h/mL)	4289.1 (778.1)	4105.0 (1258.7)
AUC 0-∞ (ng h/mL)	4435.1 (883.7)	4232.0 (1242.29)
Tmax (h)		
Median		1.5
Minimum		1.0
Maximum		3.0
MRT (h)	3.34 (0.73)	4.5 (0.76)
Cl (L/h)	14.52 (3.15)	
Vd (L)	44.6 (7.28)	

## Data Availability

Not applicable.

## Appendix A

The Revival Project Consortium:

Graciela Cárdenas ^1^, María Chávez-Canales ^2^, Ana María Espinosa ^3^, Antonio Jordán-Ríos ^2^, Daniel Anica Malgón ^3^, Manlio Fabio Márquez Murillo ^2^, Laura Victoria Torres Araujo ^1^, Ricardo Leopoldo Barajas Campos ^1^, Rosa María Wong ^4^, Luis Esteban Ramírez González ^5^, Karent Ibet Crescencio Villamil ^5^, Enrique García Velazquez ^5^, Mariana Rodríguez de la Cerda ^5^, Yoana Leyva ^2^, Joselin Hernández-Ruiz ^3^, María Luisa Hernán dez-Medel ^3^, Mireya León-Hernández ^3^, Karen Medina Quero ^6^, Anahí Sánchez Monciváis ^6^, Sergio Hernández Díaz ^6^, Ignacia Rosalía Zerón Martínez ^6^, Adriana Martínez-Cuazitl ^6^, Iván Noé Martínez Salazar ^6^, Eduardo Beltrán Sarmiento ^6^, Aldo Figueroa Peña ^6^, Patricia Saraí Hernández Hernández ^6^, Rafael Ignacio Aguilar Reynoso ^6^, Daniela Murillo Reyes ^6^, Luis Rodrigo del Río Ambriz ^6^, Rogelio Antonio Alfaro Bonilla ^6^, Jocelyn Cruz ^1^, Leonor Huerta ^7^, Nora Alma Fierro ^7^, Marisela Hernández ^7^, Mayra Pérez-Tapia ^8^, Gabriela Meneses ^9^, Helgi Jung ^10^, Erick Espíndola-Arriaga ^7^, Alberto Chinney ^5^, Sergio Rosales Mendoza ^11^, Gabriela Rosas ^12^, Juan Alberto Hernández-Aceves ^7^, Jaquelynne Cervantes-Torres ^13^, Roxana Olguin Alor ^7^, Sandra Ortega Francisco ^7^, Anai Fuentes Rodríguez ^7^, Hugo Besedovsky ^14^, Marta C. Romano ^15^, Raúl J. Bobes ^7^, Gloria Soldevila ^7^, Juan López Alvarenga ^16^, Gladis Fragoso ^7^, Juan Pedro Laclette ^7^ and Edda Sciutto ^7^

1. Departamento de Neurología, Instituto Nacional de Neurología y Neurocirugía (INNN), Av. Insurgentes Sur 3877, La Fama, Tlalpan, Ciudad de Mexico 14269, Mexico; gracielacardenas@yahoo.com.mx (G.C.); lau.torresaraujo@gmail.com (L.V.T.A.); drbarajas@hotmail.es (R.L.B.C.); alder_30@hotmail.com (A.C.H.)
2. Instituto Nacional de Cardiología Ignacio Chávez and Instituto de Investigaciones Biomédicas, Universidad Nacional Autónoma de Mexico, Juan Badiano No. 1, Col. Sección XVI, Tlalpan, Ciudad de Mexico 14080, Mexico; maria@iibiomedicas.unam.mx (M.C.-C); ajordanrios@gmail.com; (A.J.-R.); manlio.marquez@gmail.com (M.F.M.M.); qfbyoana@gmail.com (Y.L.)
3. Hospital General de Mexico Dr. Eduardo Liceaga, Dr. Balmis 148, Doctores, Cuauhtémoc, Ciudad de Mexico 06720, Mexico; Unidad de Investigación UNAM-INC; anaesga@hotmail.com (A.M.E.); anicamalagon@yahoo.com.mx (D.A.M.); hernandezjoselin@hotmail.com (J.H.R.); leonhmireya@gmail.com (M.L.H.M.)
4. Facultad de Medicina, Universidad Nacional Autónoma de Mexico, Ciudad de Mexico 04510, Mexico; rmwong@unam.mx
5. Unidad Temporal COVID-19, Centro Citibanamex. Avenida del Conscripto 311, Lomas de Sotelo, Miguel Hidalgo 11200, CDMX, Mexico; luisest01@hotmail.com (L.E.G.R.); karentibet.v@gmail.com (K.I.C.); quiqui7@hotmail.es (E.G.V.); mariana.rdlc@gmail.com (M.R.C.); a.chinney.h@gmail.com (A.C.H.)
6. Hospital Militar, Secretaría de la Defensa Nacional Periférico Blvrd Manuel Ávila Camacho s/n, Militar, Miguel Hidalgo, Ciudad de Mexico 11200, CDMX, Mexico; kmq.kmq5@gmail.com (Q.M.A.); amoncivais11@gmail.com; sehedi@yahoo.com.mx (S.H.D.); rosaliazeron_1981@hotmail.com (A.R.Z.M.); adyta0@hotmail.com (AM-C.); drivanmartinez@icloud.com (I.N.M.S.); edherzliche@hotmail.com (E.B.S.); afp_red01@hotmail.com (A.F.P.); sara.delarosa@gmail.com(P.S.H.H.); rafael.aguilar@gmail.com (R.I.A.R.); danimure95@gmail.com (D.M.R.); rodrigodlrio@gmail.com (L.R.R.A.); alfarobonillarogelioantonio@gmail.com (R.A.A.B.)
7. Departamento de Inmunología, Instituto de Investigaciones Biomédicas, Universidad Nacional Autónoma de Mexico, Ciudad de Mexico 04510, Mexico; cruz.joss0308@gmail.com (J.C.); leonorhh@iibiomedicas.unam.mx (L.H.); noraalma@iibiomedicas.unam.mx (N.A.F.); marysel_01@yahoo.com.mx (M.H.); eespindola@iibiomedicas.unam.mx (E.E.-A.); dragg412@gmail.com (J.A.H.A.); rolguinalor@iibiomedicas.unam.mx (R.O.A.); andraamyris@iibiomedicas.unam.mx (S.O.F.); afuentes@iibiomedicas.unam.mx (A.F.R.); rbobes@iibiomedicas.unam.mx (R.J.B.); soldevi@unam.mx (G.S.); gladis@unam.mx (G.F.); laclettejp@gmail.com (J.P.L.); edda@unam.mx (E.S.)
8. Unidad de Desarrollo e Investigación en Bioprocesos, Escuela Nacional de Ciencias Biológicas del Instituto Politécnico Nacional, Prolongación de Carpio y Plan de Ayala S/N, Col. Casco de Santo Tomas, Del. Miguel Hidalgo, Ciudad de Mexico 11340, Mexico; smpt.2011@hotmail.com (M.T.-P.)
9. Instituto de Diagnóstico y Referencia Epidemiológicos Dr. Manuel Martínez Báez, Francisco de Miranda 177, Lomas de Plateros, Álvaro Obregón, Ciudad de Mexico 01480, Mexico; quilaz@yahoo.com (G.M.)
10. Facultad de Química, Universidad Nacional Autónoma de Mexico, Ciudad de Mexico 04510, Mexico; helgi@unam.mx (H.J.)
11. Universidad Autónoma de San Luis Potosí, Álvaro Obregón 64, Col. Centro, C.P. 78000, San Luis Potosí, San Luis Potosí, Mexico; rosales.s@uaslp.mx (S.R.M.)
12. Facultad de Medicina, Universidad Autónoma del Estado de Morelos, Avenida Universidad No. 1001, Chamilpa, Cuernavaca 62209, Morelos, Mexico; gabyrosas62@hotmail.com (G.R.)
13. Unidad de Investigación, Facultad de Medicina Veterinaria y Zootecnia, Universidad Nacional Autónoma de Mexico, Ciudad de Mexico 04520, Mexico; paque81@hotmail.com (J.T.-C.)
14. Institute of Physiology and Pathophysiology, Emil-Mannkopff-Straße 2 u, 35037 Marburg, Germany; besedovs@staff.uni-marburg.de (H.B.)
15. Departamento de Fisiología, Biofísica y Neurociencias, Centro de Investigación y Estudios Avanzados del Instituto Politécnico Nacional, Av. Instituto Politécnico Nacional 2508, San Pedro Zacatenco, Gustavo A. Madero, Ciudad de Mexico 07360, Mexico; mromano@fisio.cinvestav.mx (M.C.R.)
16. University of Texas Rio Grande Valley—UTRGV, 1201 W University Dr, Edinburg, TX 78539, USA; juan.lopezalvarenga@utrgv.edu (J.A.L.)
